# Supplementary material for: Policy-driven transformation of global solar PV supply chains and resulting impacts
Source: Nat Commun. 2025 Jul 22;16:6742. doi: 10.1038/s41467-025-61979-5 (PMC12284120; doi:10.1038/s41467-025-61979-5)
Supplement: Supplementary file 1 — Supplementary Information [file 41467_2025_61979_MOESM1_ESM.pdf]

Supplementary Information for

Policy-driven transformation of global solar PV supply chains and resulting impacts

Can Cui[1,2], Katherine Emma Lonergan[1,2], Giovanni Sansavini[1,2,*]

[1]Institute of Energy and Process Engineering, ETH Zurich, 8092 Zurich, Switzerland

[2]Reliability and Risk Engineering, ETH Zurich, 8092 Zurich, Switzerland

*Corresponding author: Giovanni Sansavini (sansavig@ethz.ch)

Supplementary Tables

Supplementary Table 1 2

Supplementary Table 2 2

Supplementary Figures

Supplementary Fig. 1 3

Supplementary Fig. 2 3

Supplementary Fig. 3 4

Supplementary Fig. 4 5

Supplementary Fig. 5 6

Supplementary Fig. 6 7

Supplementary Fig. 7 8

Supplementary Fig. 8 9

Supplementary Fig. 9 10

Supplementary Fig. 10 11

Supplementary Fig. 11 12

Supplementary Fig. 12 13

Supplementary Fig. 13 14

Supplementary Fig. 14 15

Supplementary Fig. 15 16

Supplementary Table 1 Capacity expansion costs for the five products in the PV supply chain by region[1,2]. Unit: USD/kW of end product.

Region	Polysilicon	Ingots	Wafers	Cells	Modules
CHE	378	85	85	60	40
CHN	196	42.5	42.5	60	40
DEU	378	85	85	60	40
IND	252	42.5	42.5	60	40
KOR	252	42.5	42.5	60	40
MYS	252	42.5	42.5	60	40
ROA	252	42.5	42.5	60	40
ROE	378	85	85	60	40
ROW	378	85	85	60	40
THA	252	42.5	42.5	60	40
USA	378	85	85	60	40
VNM	252	42.5	42.5	60	40

Supplementary Table 2 Jobs in the 2023 PV supply chain[3] and projected annual growth to 2030 based on our model. Unit: full-time jobs per year. Year-over-year growth rate indicated in parentheses.

Region	2023 jobs: IRENA[3]	Annual growth by 2030: Baseline	Annual growth by 2030: Maximize European jobs
China	2,370,000	688,460 (29%)	653,633 (28%)
Europe	43,000	58,626 (136%)	85,049 (198%)
United States	33,534	41,436 (124%)	41,473 (124%)
Malaysia	43,000	2,356 (5%)	2,374 (6%)
Vietnam	78,750	4,499 (6%)	4,447 (6%)

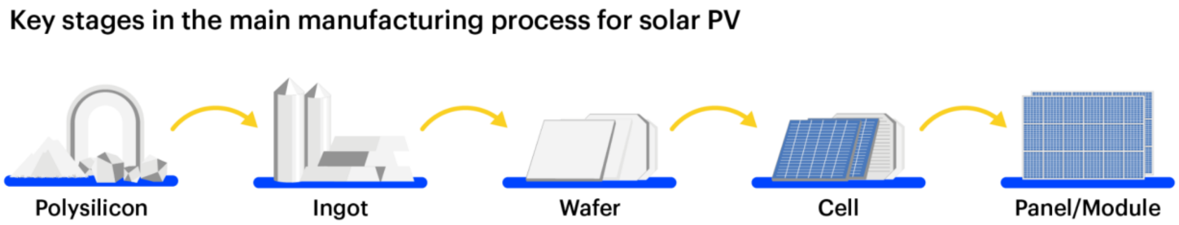

Supplementary Fig. 1 Five components of the supply chain of solar PV listed from upstream to downstream product (left-to-right). Source: IEA (2022)[4], Solar PV Global Supply Chains, License: CC BY 4.0.

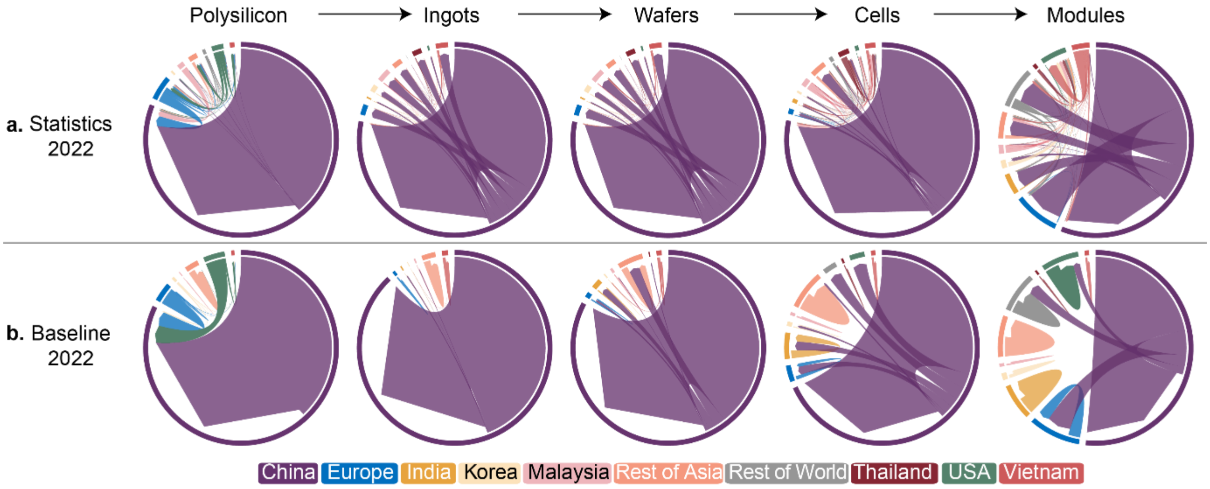

Supplementary Fig. 2 Global PV supply chain in 2022 : (a) based on statistics[5–7] and (b) based on model baseline results. The circles show the total global trade flow of the PV components, with the size of each arrow indicating the flow from exporters to importers and the colors representing individual regions. The model results are similar to the current supply chain in terms of major suppliers, but there is still space for the current supply chain to transition to the one with global economic costs and job creation optimized. The model simplifies regional strategies to achieve a globally optimized solution, which enables us to explore cost-and-job-optimal supply chain transitions under policy scenarios and assess trade-offs. However, it does not capture the full complexity of individual companies' actions or country-specific policies and results should not be seen as projections.

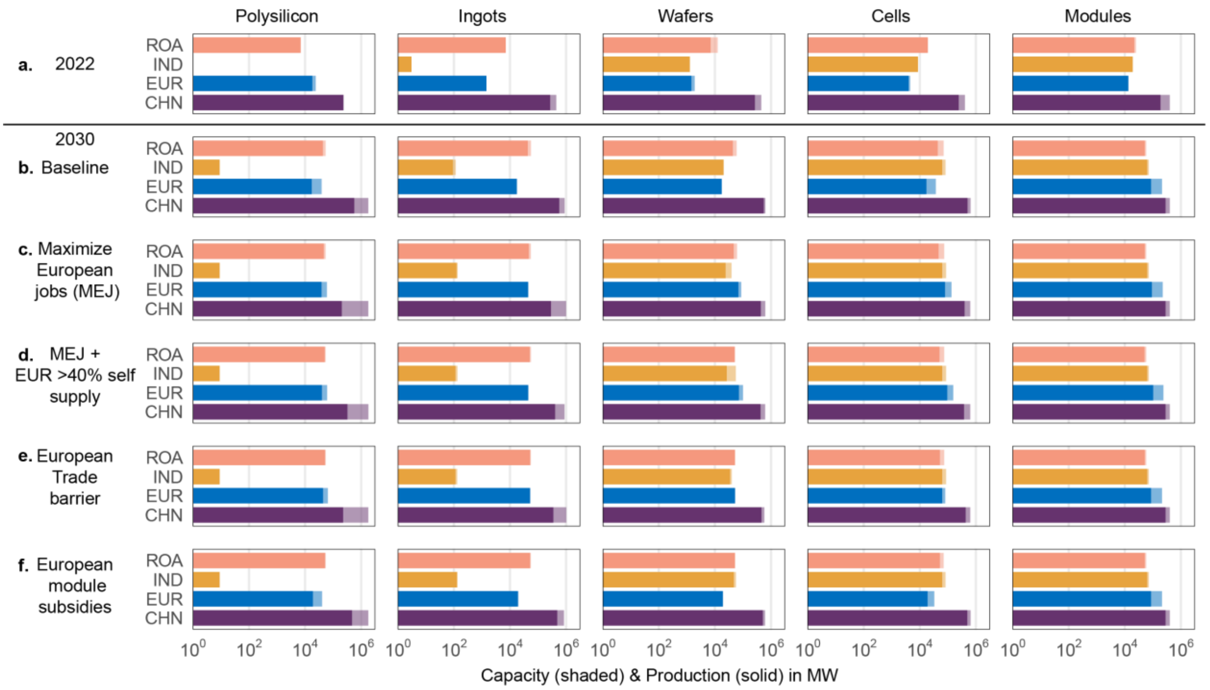

Supplementary Fig. 3 Manufacturing capacity and production by product and region: (a) in 2022, and (b–f) in 2030 under different scenarios. The 2030 scenarios include: (b) baseline, (c) maximize European jobs (MEJ), (d) maximize European jobs while meeting self-sufficiency goals, (e) introduce trade barriers against China, and (f) provide subsidies for European PV modules. Abbreviations: ROA refers to Rest of Asia, IND refers to India, EUR refers to the region including Europe, and CHN refers to mainland China. China has a surplus capacity because it supplies other regions when they cannot meet their own demand because building new capacity in those regions takes longer than China.

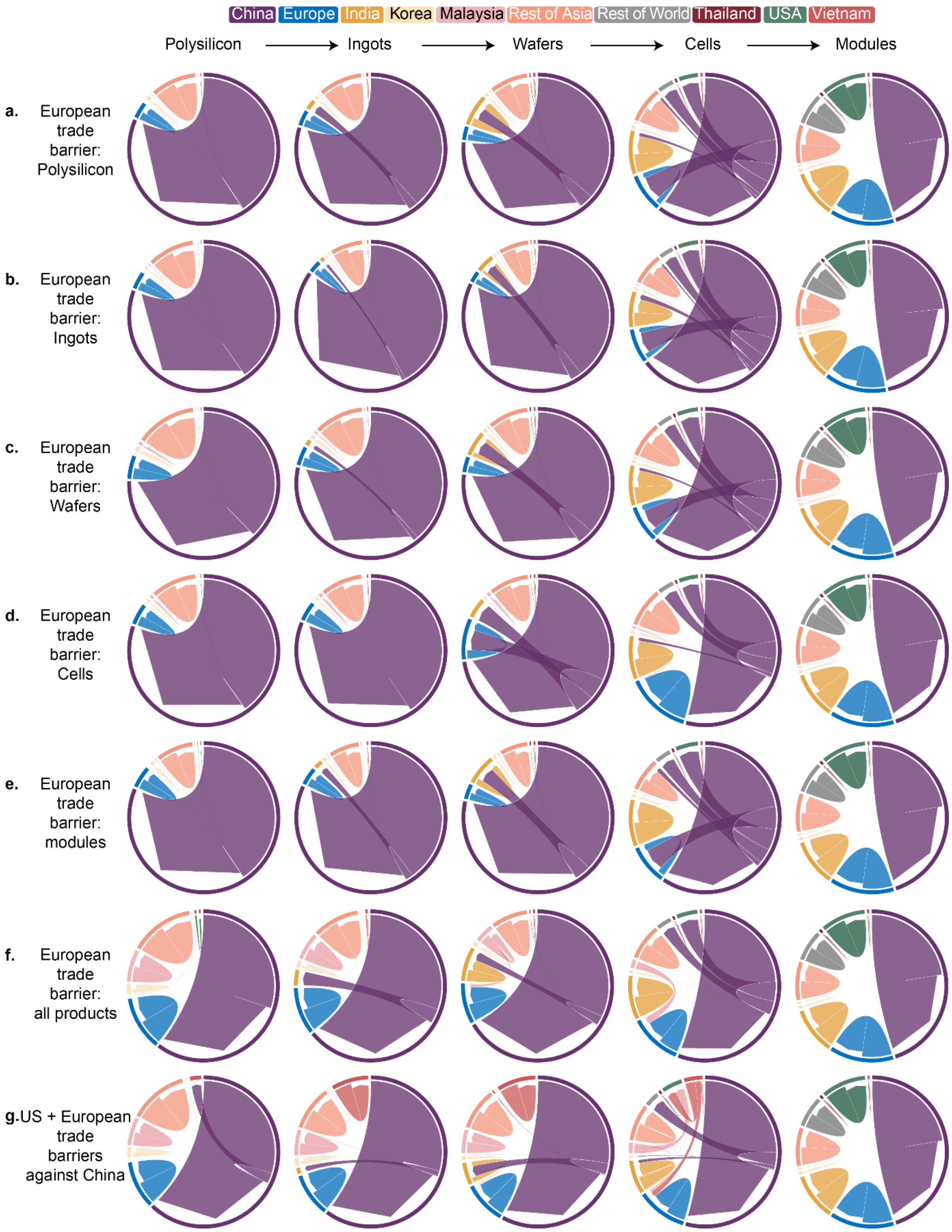

Supplementary Fig. 4 Global PV supply chain in 2030, showing trade flows by product and scenario. Panels (a–e) show the impact of introducing European trade barriers against China on individual products: polysilicon (a), ingots (b), wafers (c), cells (d), and modules (e). Panel (f) shows trade barriers on all products, and panel (g) shows both Europe and the US imposing trade barriers on all products against China. Imposing a European trade barrier with China shifts Europe's dependence from China to Malaysia, thus not totally achieving the goal of increased self-sufficiency but merely transferring it to another country. Similar effects can be expected if both the USA and Europe implement trade barriers against China – the unmet demand turns to India, Malaysia, Vietnam, and ROA. Besides, banning trade

on downstream products raises the dependence of upstream products on other regions, e.g., banning module imports leads to more imports of cells, and banning cell imports leads to more imports of wafers.

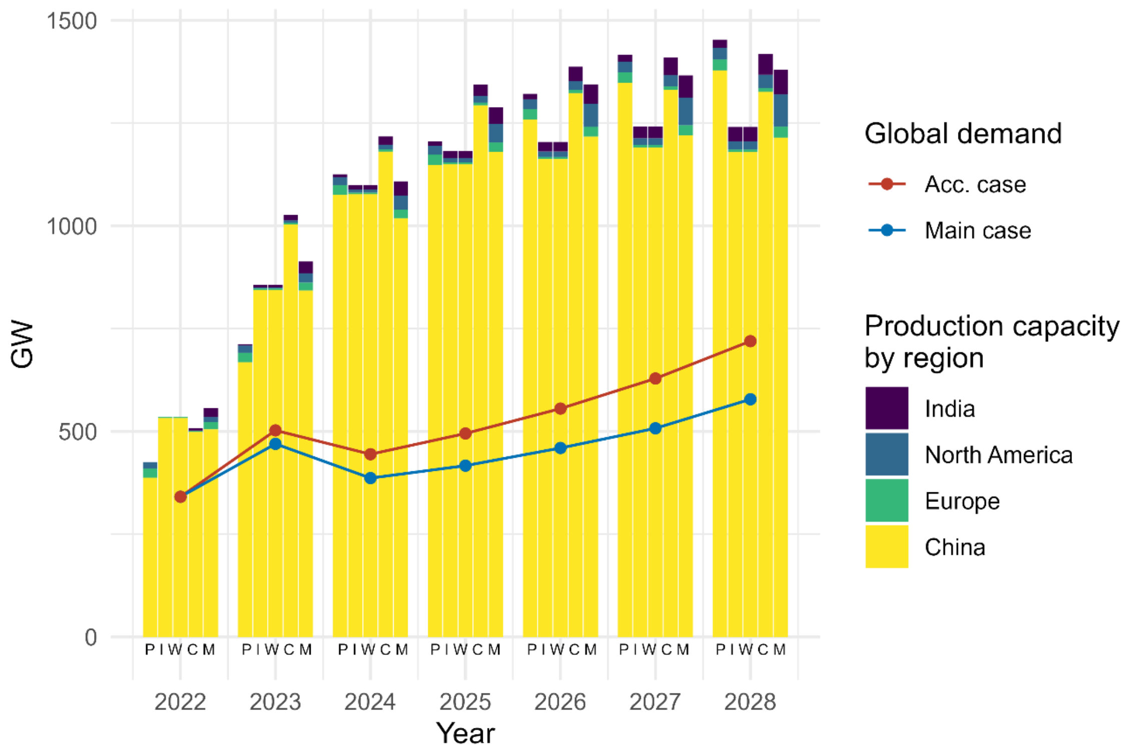

Supplementary Fig. 5 Historical global manufacturing capacity of polysilicon (P), ingots (I), wafers (W), cells (C), and modules (M) by region versus global demand according to the International Energy Agency[8]. The IEA considers in the main case and accelerated (Acc. case) cases over 2022-2028 (ingots and wafers are assumed to share identical capacities)[8].

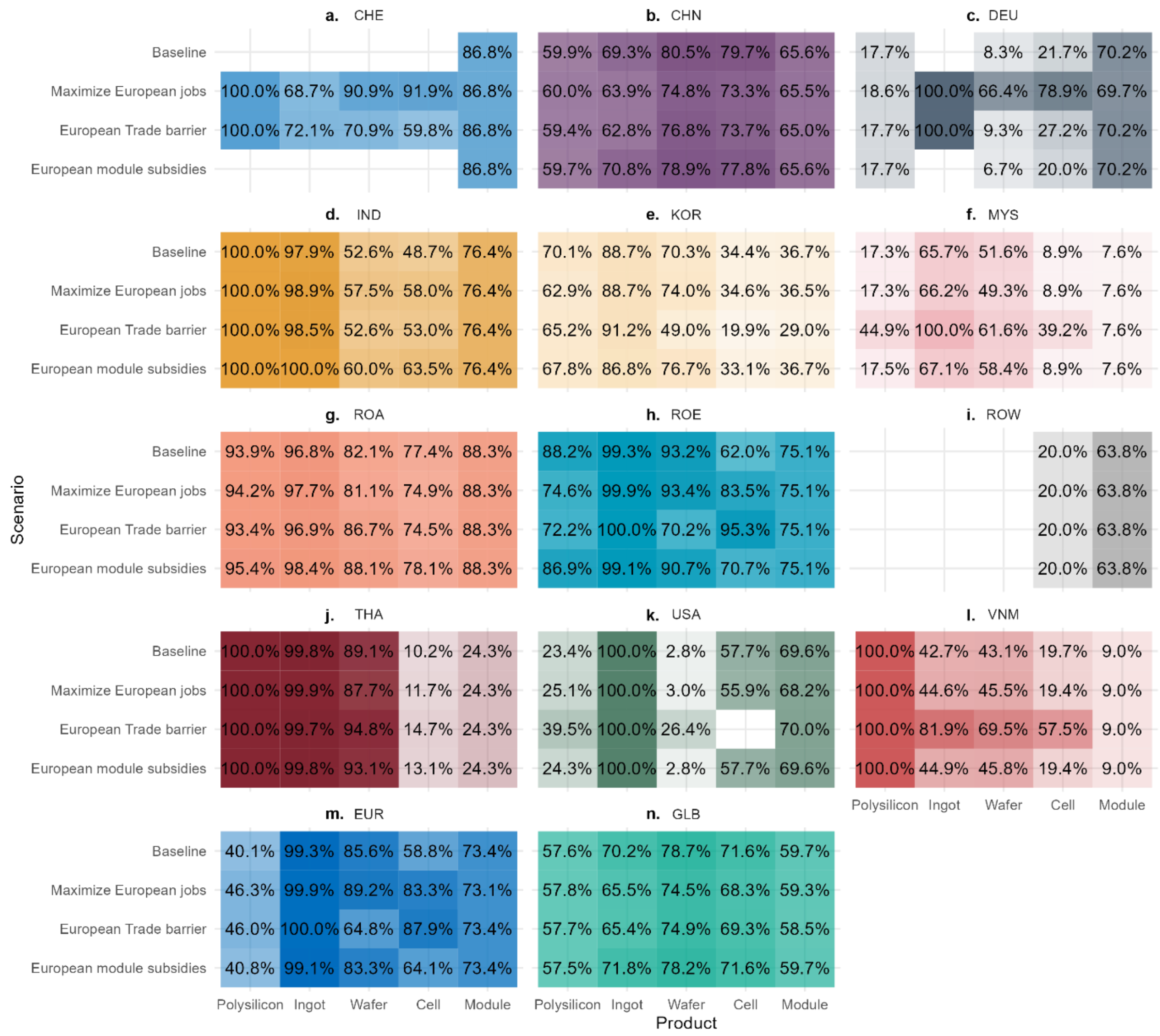

Supplementary Fig. 6 Average efficiency of manufacturing capacity of the supply of PV products over 2021-2030 by scenario, product and region. Panels (a–n) show regional results: (a) Switzerland (CHE), (b) mainland China (CHN), (c) Germany (DEU), (d) India (IND), (e) Korea (KOR), (f) Malaysia (MYS), (g) Rest of Asia (ROA), (h) Rest of Europe (ROE), (i) Rest of World (ROW), (j) Thailand (THA), (k) United States (USA), (l) Viet Nam (VNM), (m) Europe (EUR, including DEU, CHE, and ROE), and (n) global total (GLB). Global capacity utilization ranges from 58-79% (as shown in panel (n)). Blank grids mean no manufacturing capacity in the region-scenario match.

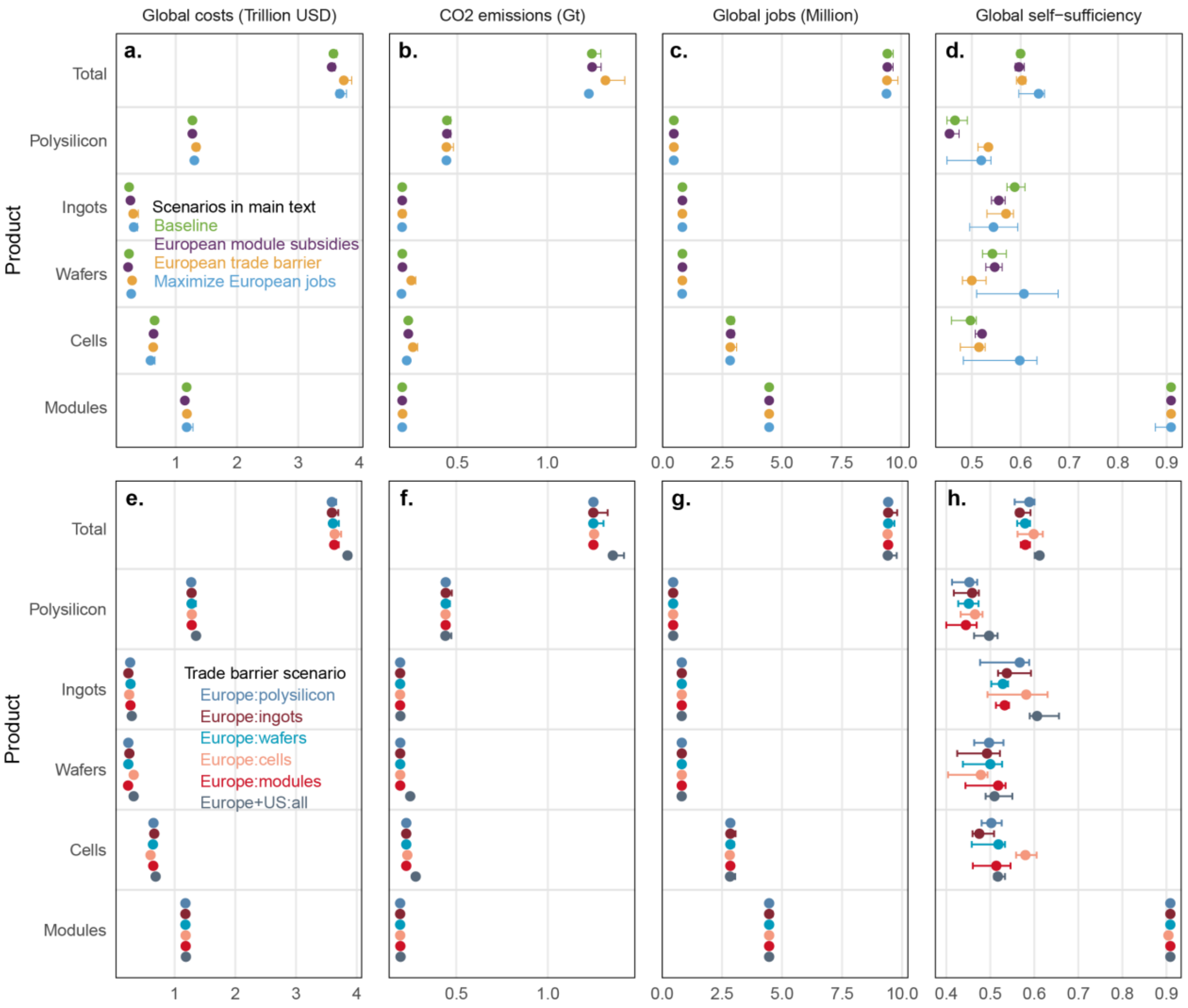

Supplementary Fig. 7 Global impacts of supply chain transformation by product and scenario. Each point shows the median, and error bars indicate the minimum–maximum range. Panels (a–d) correspond to the scenarios in Fig. 3 of the main text: baseline, subsidies for European PV modules, trade barriers against China, and maximizing European jobs. Panels (e–h) show additional trade barrier scenarios, including product-specific trade barriers against China and a full trade ban by both Europe and the US. Colors represent different scenarios. Impacts are listed in terms of global cumulative costs (a and e), cumulative carbon emissions (b and f), cumulative job creation (c and g), and average self-sufficiency of supply (d and h). Among the components, polysilicon is the most expensive product, while module supply creates the most jobs with the highest self-sufficiency. Trade barriers can influence self-sufficiency with economic and environmental costs.

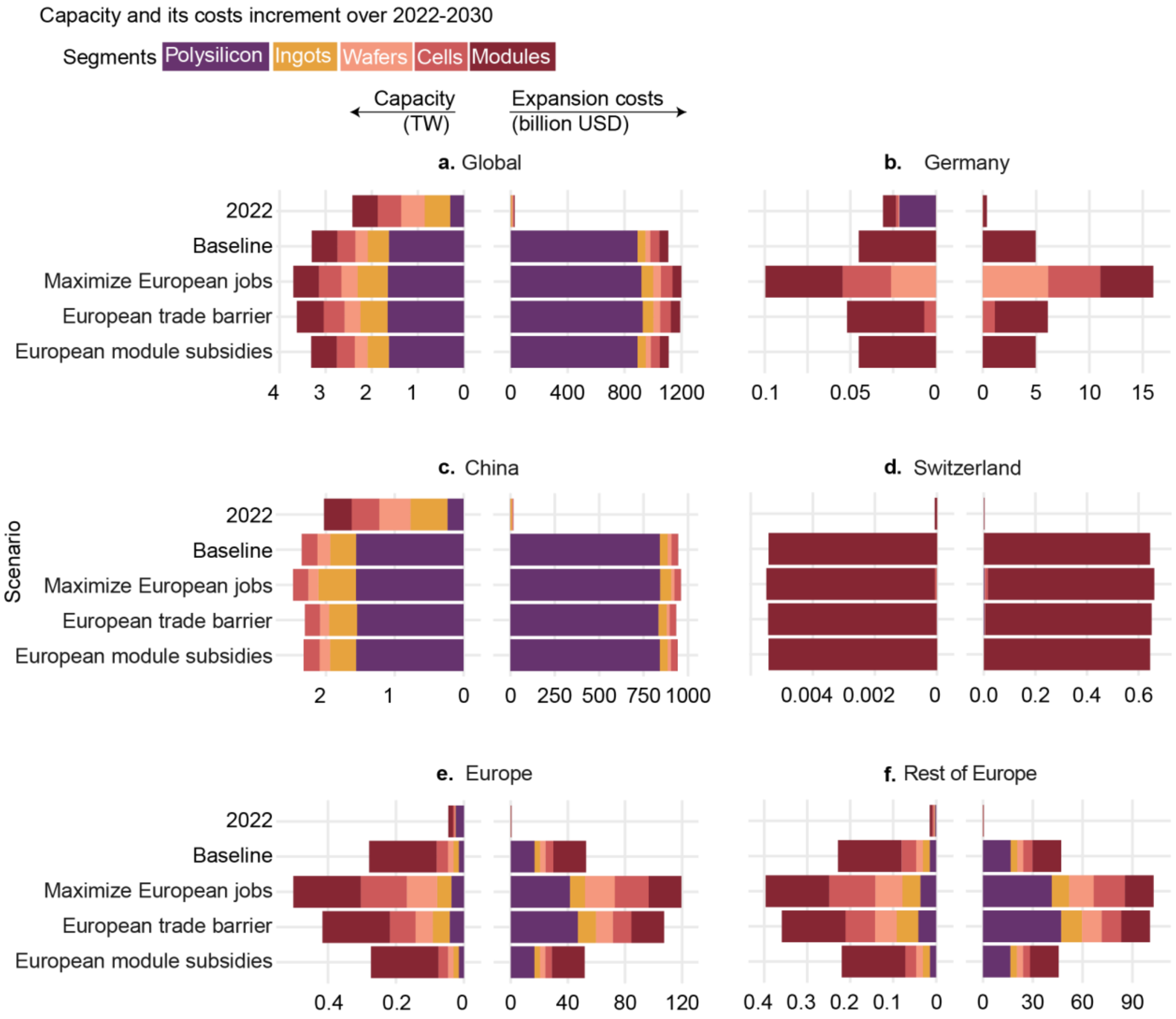

Supplementary Fig. 8 Manufacturing capacity expansion and associated costs to close the gap between 2022 and 2030, by region, product, and scenario. Panels show results for: (a) global total, (b) Germany, (c) China, (d) Switzerland, (e) Europe (including Germany, Switzerland, and Rest of Europe), and (f) Rest of Europe. Capacity expansion is listed on the left in terawatt hours and expansion costs are written on the right in billions of US dollars.

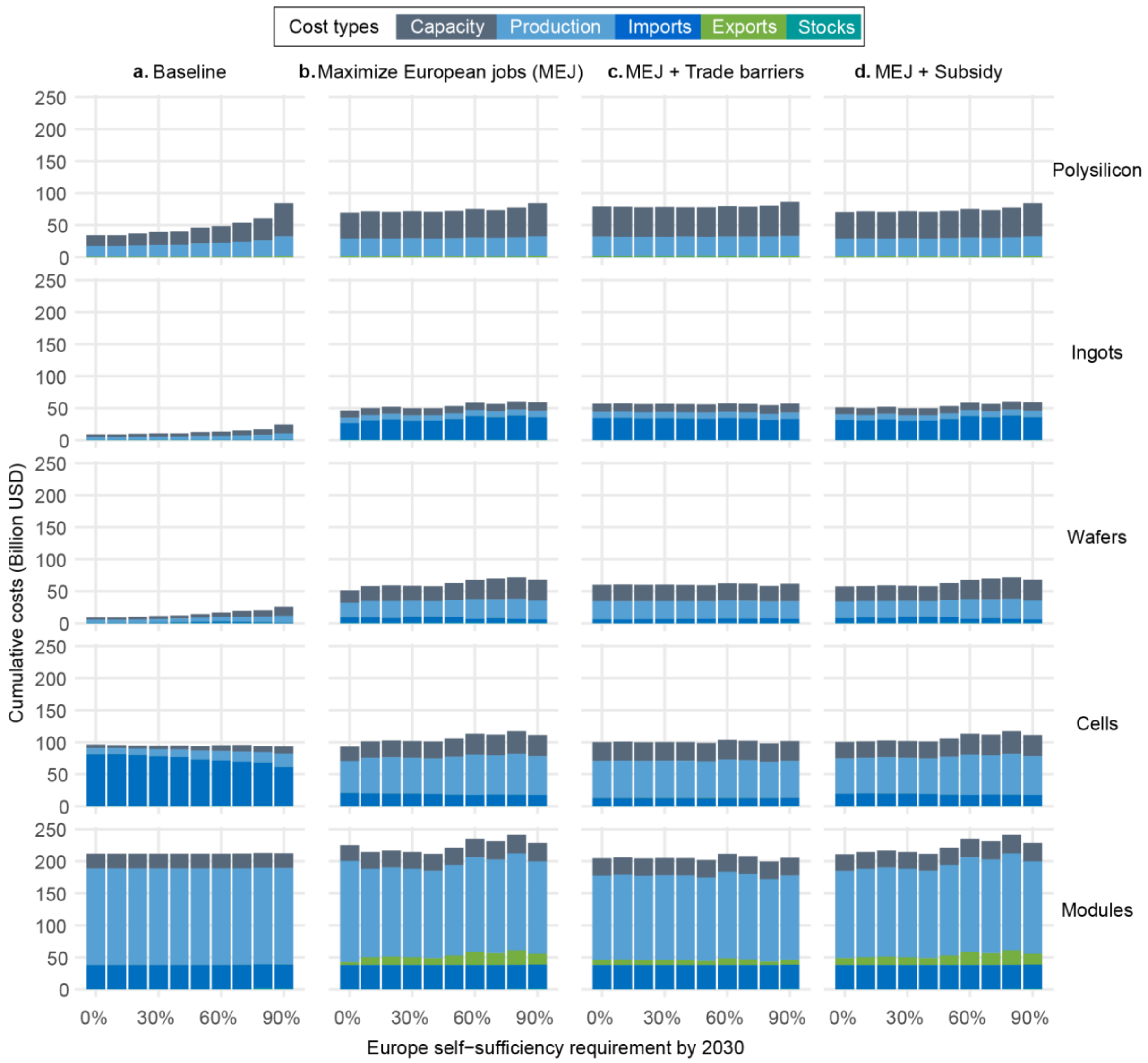

Supplementary Fig. 9 Europe cumulative industry costs and self-sufficiency requirements for each product by 2030, by scenario and cost type in colors. Scenarios include: (a) baseline, (b) maximizing European jobs, (c) introducing trade barriers against China while maximizing European jobs, and (d) providing subsidies for European PV modules while maximizing European jobs.

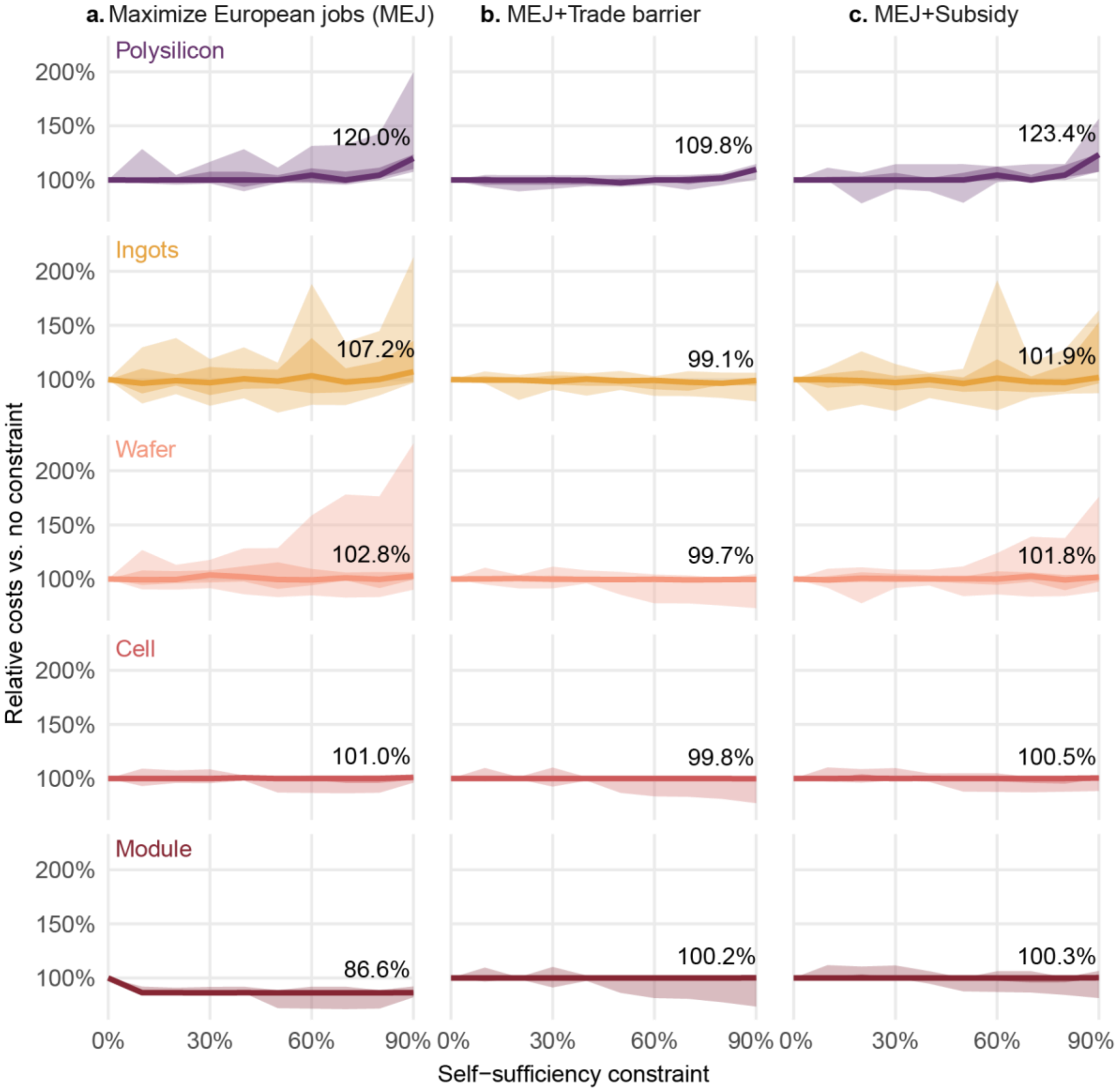

Supplementary Fig. 10 Changes in Europe's costs, expressed as a percentage, under 0-90% self-sufficiency constraints compared to no self-sufficiency constraints, by product and scenario. Lines show the median values; shaded areas with light colors show the min-max range and the deeper-color shaded areas show the 66% confidence intervals. Scenarios include: (a) maximizing European jobs, (b) introducing trade barriers against China while maximizing European jobs, and (c) providing subsidies for European PV modules while maximizing European jobs.

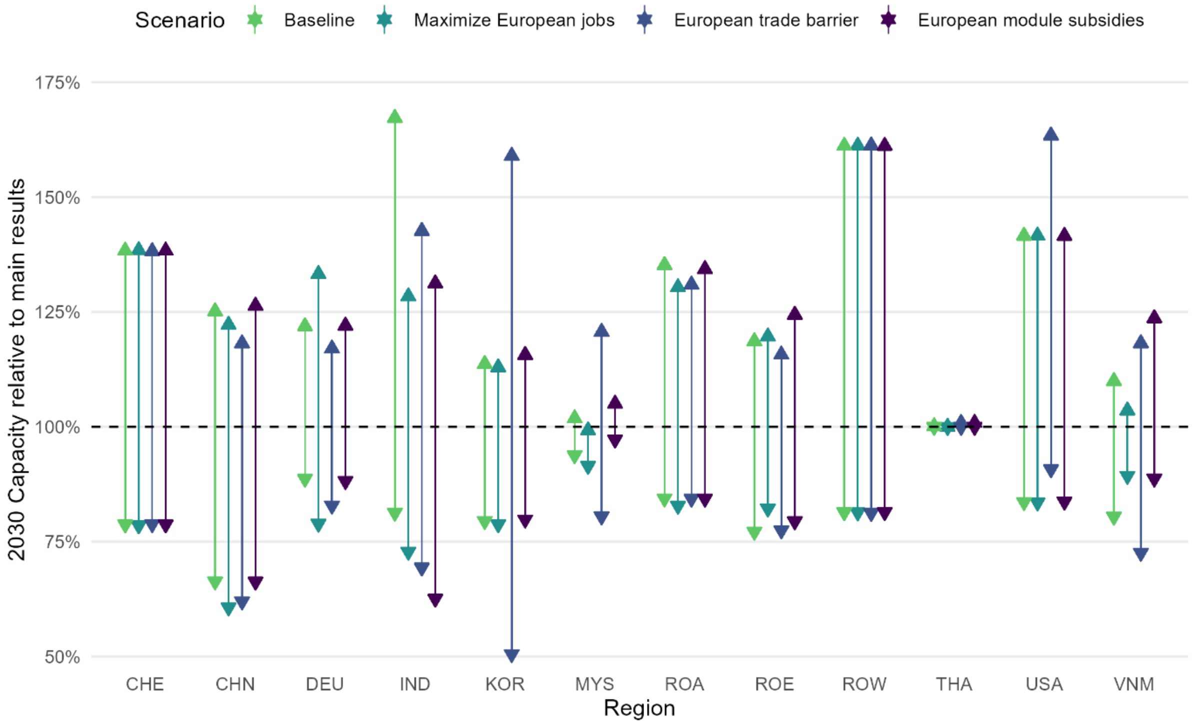

Supplementary Fig. 11 2030 manufacturing capacity ranges (95% confidence interval) by scenario and region considering demand uncertainty. Abbreviations: CHE refers to Switzerland, CHN refers to mainland China, DEU refers to Germany, IND refers to India, KOR refers to Korea, MYS refers to Malaysia, ROA refers to Rest of Asia, ROE refers to Rest of Europe, ROW refers to Rest of World, THA refers to Thailand, USA refers to the United States, and VNM refers to Viet Nam.

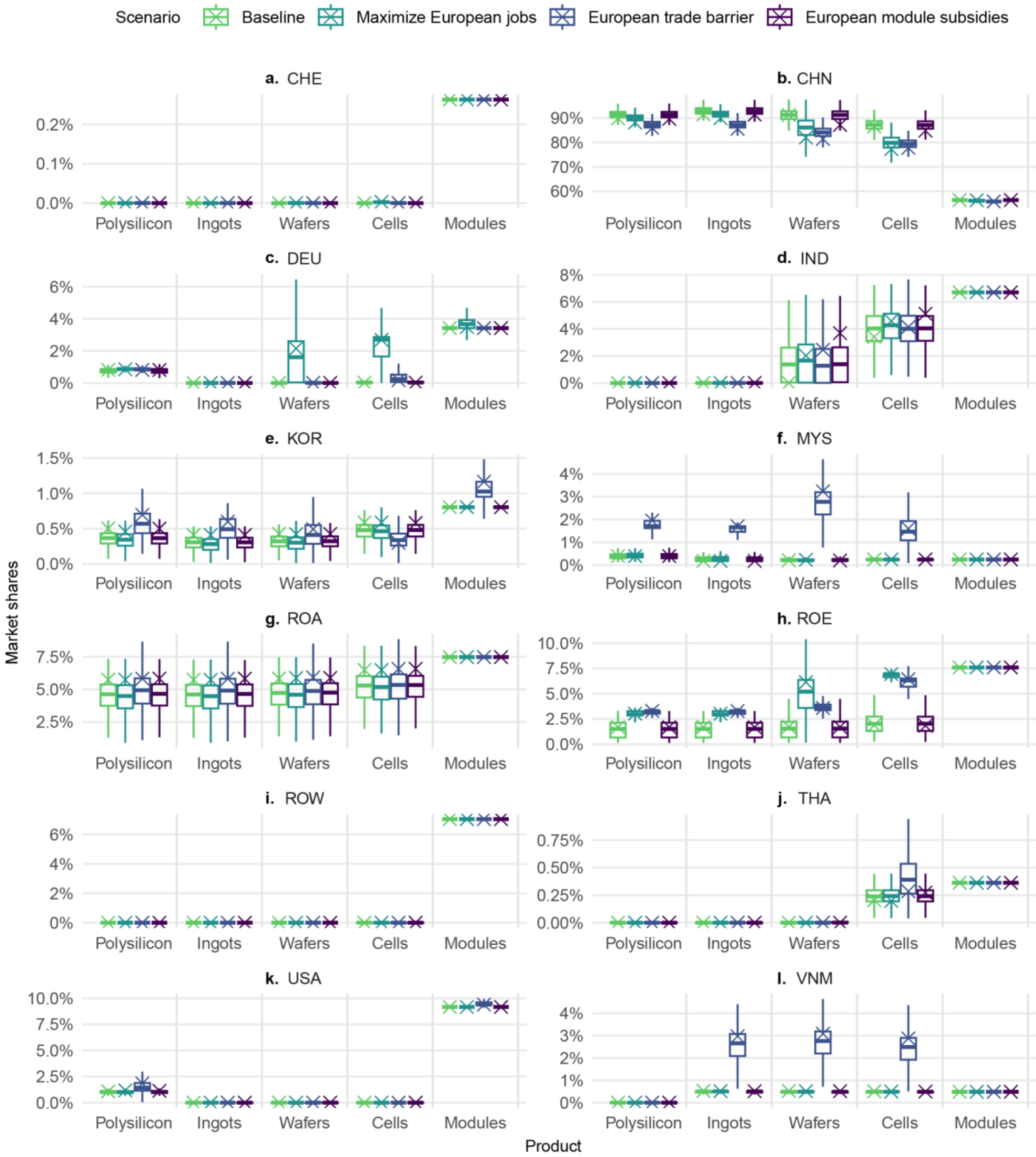

Supplementary Fig. 12 Regional market shares by scenario considering uncertain costs and demand. The boxplot shows the median as the center line, the upper and lower quartiles as the box limits (middle 50%), and whiskers extending up to 1.5 times the interquartile range beyond the quartiles. The symbols "×" label the main results presented in the paper. Panels show results for: (a) Switzerland (CHE), (b) mainland China (CHN), (c) Germany (DEU), (d) India (IND), (e) Korea (KOR), (f) Malaysia (MYS), (g) Rest of Asia (ROA), (h) Rest of Europe (ROE), (i) Rest of World (ROW), (j) Thailand (THA), (k) United States (USA), and (l) Viet Nam (VNM).

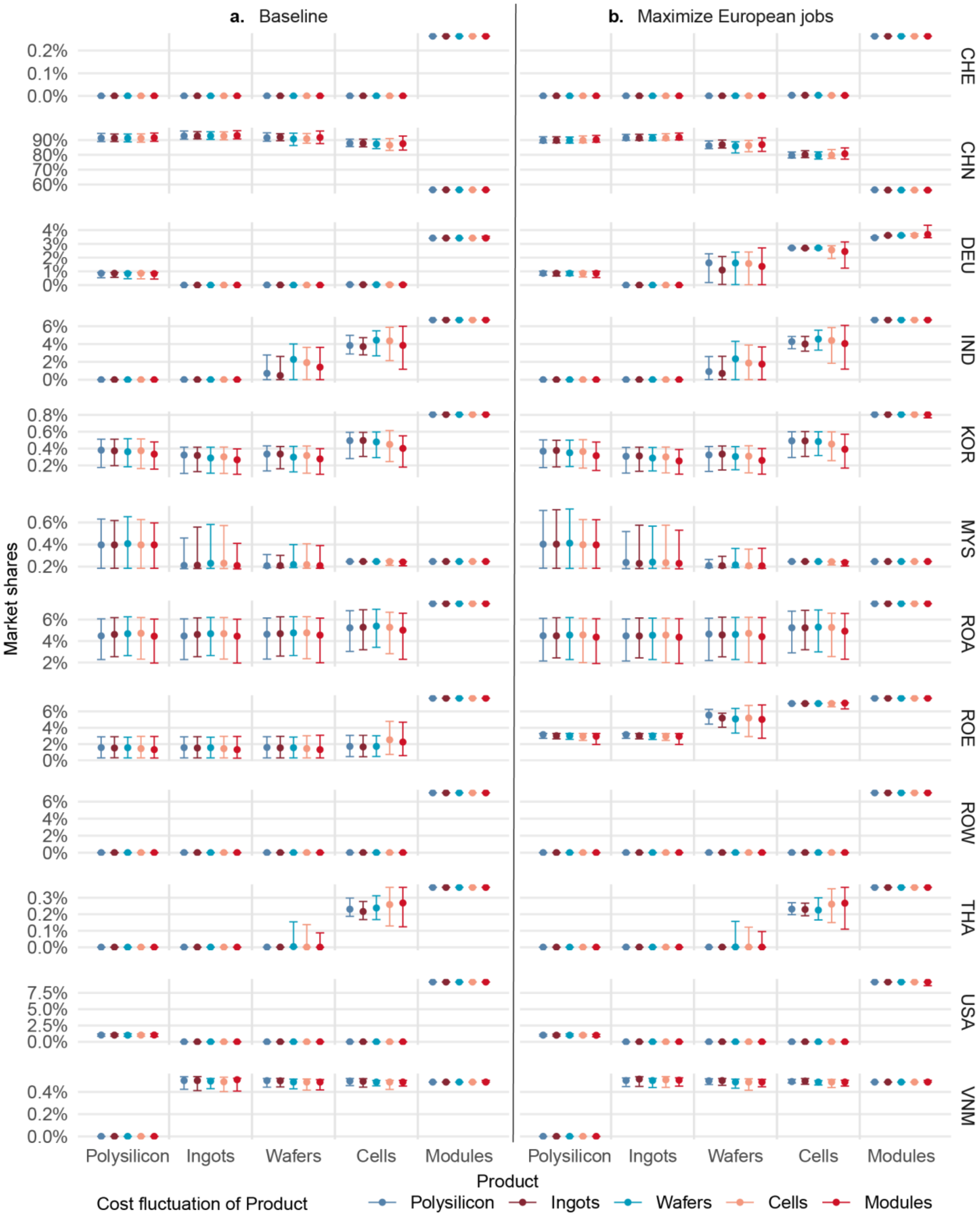

Supplementary Fig. 13 Regional market shares by product under two scenarios: (a) baseline and (b) maximizing European jobs. Results account for per-product production cost fluctuations. The points show the median and the error bars show the range of 95% confidence interval. The cost fluctuation of individual products does not make significant difference on the regional shares, although downstream products' cost fluctuations have a bigger impact on market share than upstream products. Abbreviations: CHE refers to Switzerland, CHN refers to mainland China, DEU refers to Germany, IND refers to India, KOR refers to Korea, MYS refers to Malaysia, ROA refers to Rest of Asia, ROE refers to Rest of Europe, ROW refers to Rest of World, THA refers to Thailand, USA refers to the United States, and VNM refers to Viet Nam.

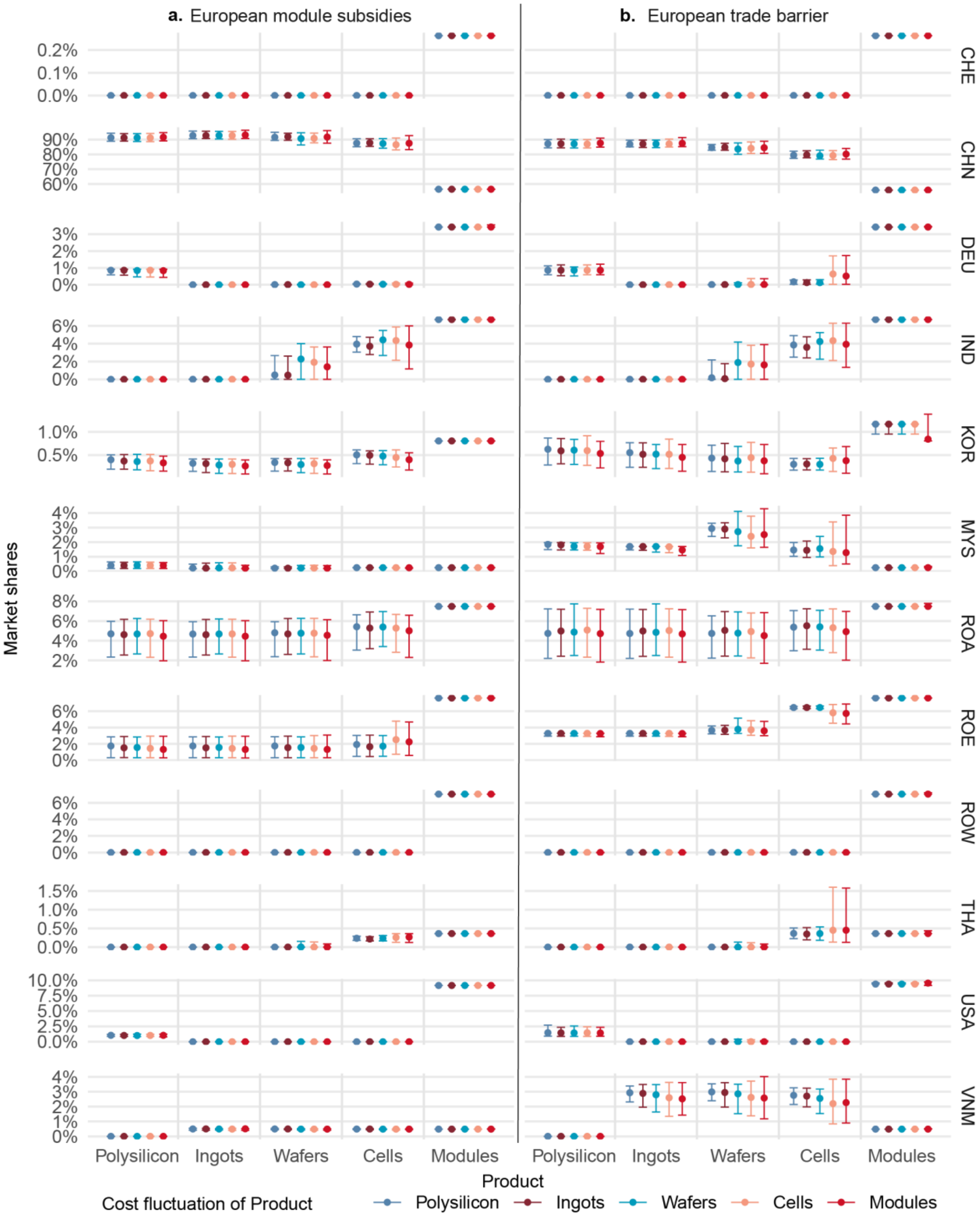

Supplementary Fig. 14 Regional market shares by product under two scenarios: (a) European module subsidies and (b) European trade barriers. Results account for per-product production cost fluctuations. The points show the median and the error bars show the range of 95% confidence interval. The cost fluctuation of individual products does not make significant difference on the regional shares, although downstream products' cost fluctuations have a bigger impact on market share than upstream products. Abbreviations: CHE refers to Switzerland, CHN refers to mainland China, DEU refers to Germany, IND refers to India, KOR refers to Korea, MYS refers to Malaysia, ROA refers to Rest of Asia, ROE refers to Rest of Europe, ROW refers to Rest of World, THA refers to Thailand, USA refers to the United States, and VNM refers to Viet Nam.

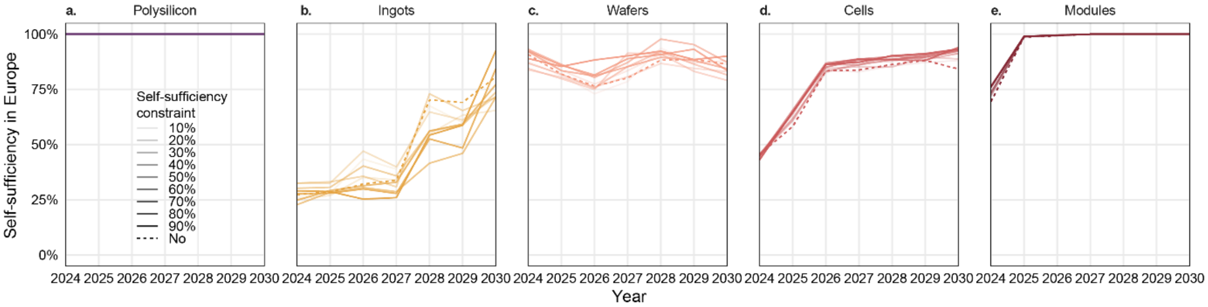

Supplementary Fig. 15 Changes in Europe's self-sufficiency by product, expressed as a percentage. Panels show results for: (a) polysilicon, (b) ingots, (c) wafers, (d) cells, and (e) modules. Various lines represent different self-sufficiency scenarios ranging from 10% to 90%, as well as a scenario with no constraint. The second step, ingot manufacturing, is the main bottleneck limiting Europe's self-sufficiency in the supply chain.

Supplementary References

1. Woodhouse, M. A., Smith, B., Ramdas, A. & Margolis, R. M. Crystalline Silicon Photovoltaic Module Manufacturing Costs and Sustainable Pricing: 1H 2018 Benchmark and Cost Reduction Road Map. http://www.osti.gov/servlets/purl/1495719/ (2019) doi:10.2172/1495719.
2. The European House - Ambrosetti. Energy Transition Strategic Supply Chains - Industrial Roadmap for Europe and Italy. (2023).
3. IRENA & ILO. Renewable Energy and Jobs: Annual Review 2024. (2024).
4. International Energy Agency. Special Report on Solar PV Global Supply Chains. (OECD, 2022). doi:10.1787/9e8b0121-en.
5. UN Comtrade. Trade Data. (2024).
6. BloombergNEF. BloombergNEF Data & Tools. (2023).
7. International Energy Agency. PVPS Trends in Photovoltaic Applications 2023. https://iea-pvps.org/wp-content/uploads/2023/10/PVPS_Trends_Report_2023_WEB.pdf (2023).
8. International Energy Agency. Renewables 2023 - Analysis and Forecast to 2028. https://iea.blob.core.windows.net/assets/96d66a8b-d502-476b-ba94-54ffda84cf72/Renewables_2023.pdf (2024).
