# Supplementary material for: Policy-driven transformation of global solar PV supply chains and resulting impacts
Source: Nat Commun. 2025 Jul 22;16:6742. doi: 10.1038/s41467-025-61979-5 (PMC12284120; doi:10.1038/s41467-025-61979-5)
Supplement: Supplementary file 2 — Description of Additional Supplementary File [file 41467_2025_61979_MOESM2_ESM.pdf]

Description of Additional Supplementary Files

Supplementary Data 1. Data used in the optimization model for the solar PV supply chain.
